# Posttraumatic stress disorder and depression of survivors 12 months after the outbreak of Middle East respiratory syndrome in South Korea

**DOI:** 10.1186/s12889-020-08726-1

**Published:** 2020-05-15

**Authors:** Hye Yoon Park, Wan Beom Park, So Hee Lee, Jeong Lan Kim, Jung Jae Lee, Haewoo Lee, Hyoung-Shik Shin

**Affiliations:** 1 Seoul National University Hospital, Seoul, Republic of Korea; 2 Seoul National University College of Medicine, Seoul, Republic of Korea; 3 National Medical Center, 245, Eulji-ro, Jung-gu, Seoul, 04564 Republic of Korea; 4 Chungnam National University School of Medicine, Daejeon, Republic of Korea; 5 Dankook University School of Medicine, Cheonan, Chungnam, Republic of Korea; 6 Seoul Medical Center, Seoul, Republic of Korea

**Keywords:** Emerging infectious diseases, Middle East respiratory syndrome, Posttraumatic stress disorder, Depression, Mental health

## Abstract

**Background:**

The 2015 outbreak of Middle East Respiratory Syndrome (MERS) in the Republic of Korea is a recent and representative occurrence of nationwide outbreaks of Emerging Infectious Diseases (EIDs). In addition to physical symptoms, posttraumatic stress disorder (PTSD) and depression are common following outbreaks of EID.

**Methods:**

The present study investigated the long-term mental health outcomes and related risk factors in survivors of MERS. A prospective nationwide cohort study was conducted 12 months after the MERS outbreak at multi-centers throughout Korea. PTSD and depression as the main mental health outcomes were assessed with the Impact of Event Scale-Revised Korean version (IES-R-K) and the Patient Health Questionnaire-9 (PHQ-9) respectively.

**Results:**

42.9% of survivors reported PTSD (IES-R-K ≥ 25) and 27.0% reported depression (PHQ-9 ≥ 10) at 12 months post-MERS. A multivariate analysis revealed that anxiety (adjusted odds ratio [aOR], 5.76; 95%CI, 1.29–25.58; *P* = 0.021), and a greater recognition of stigma (aOR, 11.09, 95%CI, 2.28–53.90; *P* = 0.003) during the MERS-affected period were independent predictors of PTSD at 12 months after the MERS outbreak. Having a family member who died from MERS predicted the development of depression (aOR, 12.08, 95%CI, 1.47–99.19; *P* = 0.020).

**Conclusion:**

This finding implies that psychosocial factors, particularly during the outbreak phase, influenced the mental health of patients over a long-term period. Mental health support among the infected subjects and efforts to reduce stigma may improve recovery from psychological distress in an EID outbreak.

## Background

The 2015 outbreak of the Middle East Respiratory Syndrome coronavirus (MERS-CoV) in the Republic of Korea had an enormous impact on medical, psychological, and social issues nationwide [1]. The outbreak lasted from May 2015 to Dec. 2015 and resulted in 186 infected patients within the initial 2 months, 16,693 officially isolated individuals, and an overall mortality of 38 patients in a total of 50 million population [2]. Acute infectious outbreaks of Emerging Infectious Diseases (EIDs) are known to influence the physical as well as the mental health of affected patients, as observed during similar events such as the Severe Acute Respiratory Syndrome (SARS) outbreak [3], which was associated with such issues during the acute phase [4] and the long-term follow-up phase [5, 6]. 35% of 425 survivors expressed anxiety or depressive symptoms at 1-month post-SARS in Hong Kong where 1755 citizens were infected, and its fatality was 17.0% [4]. In ninety survivors’ cohort study for 30 months in Hong Kong, post-SARS cumulative incidence of psychiatric disorders was 58.9%. The most common diagnoses were PTSD (25.6%) and depression (13.3%) [5].

Few studies have investigated the psychological impact of the 2015 Korean MERS-CoV outbreak, but a survey conducted during this period found that 90% of the general public reported worrying about being infected by MERS-CoV and that 46% of this population experienced psychological distress [7]. Another study reported that 7.6% of 1656 isolated individuals exhibited anxiety symptoms and that 16.6% of this group reported feelings of anger during the isolation period [8]. In contrast, anxiety was present in 47.2% of MERS patients [8], which was more prevalent than the rate of anxiety in isolated people without the MERS-CoV infection. Compared to patients with other diseases, those with EIDs may experience greater suffering in terms of the physical and psychiatric symptoms of the infectious illness itself [9]; extreme fear and anxiety due to their unfamiliarity with the disease, which may be life-threatening [10]; abrupt isolation from family and society during the illness [8]; stigma due to the infectious disease [11]; the unexpected death of a family member; and/or social impairments [12].

Given that some factors, such as grief or stigma, may be persistent following the MERS illness, the suffering of afflicted individuals may negatively influence recovery in their daily lives during the acute outbreak period as well as the post-MERS period. Studies of SARS survivors in Hong Kong and China reported persistent psychological burdens, including post-traumatic stress disorder (PTSD), in over 40% of the survivors after 3 years [13]. However, no studies have investigated the mental health status of MERS survivors. Thus, the present study explored mental health issues and related factors in MERS survivors 12 months after the outbreak to determine the long-term psychological outcomes of this population.

## Methods

### Study design

**Fig. 1:**
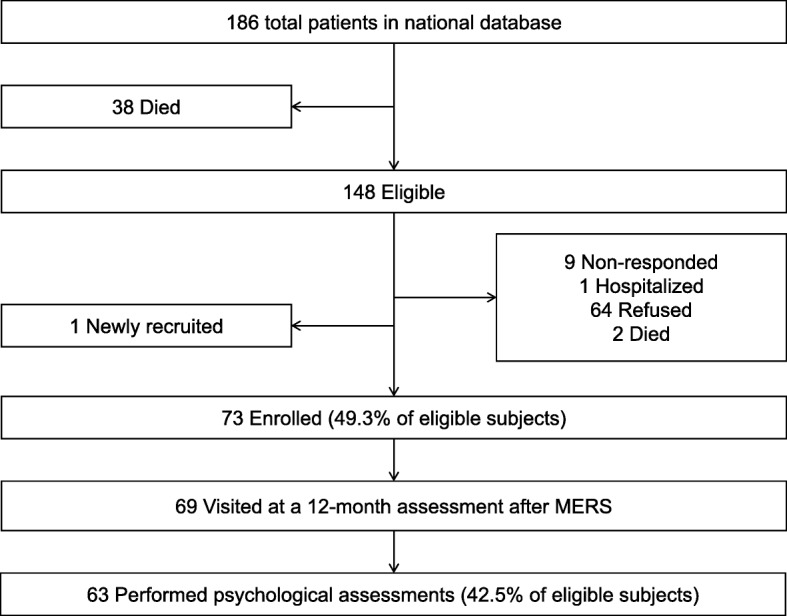
Recruitment of participants in survivors of the MERS outbreak in South Korea

The present study was part of a prospective nationwide cohort study of MERS survivors conducted at multi-centers in the Republic of Korea. For purposes of this study, a MERS survivor was defined as a patient who was diagnosed with the MERS-CoV infection and then completely recovered, as confirmed by the Korean government during the 2015 outbreak. Of 148 MERS survivors who were eligible, 73 consented to participate in the study initially when they were contacted by phone and mail at 6 months post-MERS (Fig. 1). Of these participants, 69 survivors completed the 12-month assessment that consisted of medical tests between June 2016 and August 2016. Among them, 63 participants provided consent to participate in the psychological assessments in five tertiary hospitals: National Medical Center, Seoul National University Hospital, Chungnam National University Hospital, Seoul Medical Center, and Dankook University Hospital. All subjects were older than 19 years of age at enrollment, voluntarily participated in the study, and answered the questionnaires independently. Written informed consent was obtained from all subjects, and the study was approved by the Institutional Review Board of each study hospital.

### Measures

All subjects responded to self-report questionnaires assessing sociodemographic characteristics, previous history of medical illness or psychiatric visit, illness experiences during the MERS-CoV infection period, and psychological features. Questions about MERS-related illness experiences solicited information regarding status during infection, duration of hospitalization, presence of pneumonia, whether a ventilator or extracorporeal membrane oxygenation was applied, and whether a family member died from MERS.

To determine psychological outcomes, PTSD was assessed with the Impact of Event Scale-Revised Korean version (IES-R-K) [14, 15], and depression was evaluated with the Patient Health Questionnaire-9 (PHQ-9) [16, 17]. The IES-R-K is a 22-item scale that assesses symptoms of intrusion, avoidance and numbing, and hyperarousal related to a particular life-threatening event (i.e., MERS-CoV infection in the present study). Each item is rated on a scale ranging from 0 to 4, and a total score ≥ 25 is considered to be clinically significant [15]. The PHQ-9 is a nine-item scale that assesses depression based on the symptoms of major depressive disorder included in the Diagnostic and Statistical Manual of Mental Disorders-fourth edition (DSM-IV) [16]. Significant depression is considered to be present when the total score is > 10 [17].

Current suicidality was assessed with the suicidality module of the Mini-International Neuropsychiatric Interview (K-MINI) [18, 19], which is composed of six weighted items rated as ‘yes’ or ‘no’: wish for death (weight of 1), wish for self-harm (weight of 2), suicidal thoughts (weight of 6), suicide plan (weight of 10), suicide attempt in the past 1 month (weight of 10), and lifetime suicide attempt (weight of 4). The suicidality score is the sum of the weighted score of the six items, and a total score ≥ 6 is considered to be above moderate degree of risk. Anxiety was assessed with the Generalized Anxiety Disorder-7 (GAD-7) scale, which consists of seven items rated using a four-point Likert scoring system [20]. A total score ≥ 10 is considered to be significant. The PHQ-9 and the GAD-7 were administered additionally at two points, before and during infection with MERS-CoV, based on participant recall.

Insomnia was evaluated with the Korean version of the Insomnia Severity Index (ISI-K) [21], a five-item measure relying on a five-point scale that assesses current sleep problems and interference with daily functioning; clinical insomnia was considered to be present if the total score was ≥15. MERS stigma was assessed with an adjusted version of the 40-item Berger Human Immunodeficiency Virus (HIV) stigma scale [22] and the 8-item short version of the HIV stigma scale [23]. This questionnaire contains eight items rated on a four-point Likert-type scale that ranges from “strongly disagree” to “strongly agree” and assesses the four domains of stigma: personalized stigma, disclosure concerns, negative self-image, and concerns with public attitudes; the Cronbach’s α in the present study was 0.919. The present study also included the Brief COPE, which is a 28-item questionnaire that uses a four-point Likert scale to measure three distinctive coping strategies: emotion-focused, problem-focused, and dysfunctional [24]. The social support systems of participants were assessed with the Medical Outcome Study Social Support Survey (MOS-SSS) [25], which includes 19 items that are scored on a scale from 0 to 100 and assesses emotional/information support, tangible support, positive social interactions, and affectionate support. To examine the impact of social support on mental health in a regression analysis, poor social support was defined as a MOS-SSS score lower than that of the 25th percentile for all participants.

### Statistical analysis

The sociodemographic characteristics, MERS-related clinical characteristics, and mental health status of the participants are presented as both numerical values and percentages. The present study placed a particular focus on PTSD and depression, which were the two most prevalent problems 12 months post-MERS in the descriptive analysis. Accordingly, the subjects were divided into two groups based on the presence of significant PTSD or depression. Independent t-tests were conducted to compare the mental health status between the two groups (*P* < 0.002, adjusted for multiple comparisons), a stepwise regression analysis was performed to identify independent risk factors for PTSD and depression at 12 months after the MERS outbreak, and a univariate analysis was used to identify potential mediating factors associated with PTSD/depression (*P* < 0.10). Subsequently, a backward multivariate logistic regression analysis was performed using variables identified as significant in the univariate analysis (*P* < 0.05). Although depression during MERS and current MERS stigma were significant in the univariate analysis, these variables were not entered into the multivariate regression analysis due to multicollinearity with anxiety during MERS (r = 0.831, *P* < 0.001) and MERS stigma during MERS (r = 0.628, P < 0.001), respectively. All data were analyzed with SPSS for Windows version 21.0 (IBM Corp.; Armonk, NY, USA) except for the multivariate logistic regression analysis, which was performed with STATA version 14.0 (STATA; College Station, TX, USA).

## Results

The demographic characteristics of the subjects are presented in Table 1. Although more male (*n* = 39, 61.9%) than female subjects were included in the study, the age distribution was relatively even (mean age: 49.2 years, standard deviation [SD]: 12.6). Of the 63 subjects, 15.9% had a history of a visit to a psychiatric clinic prior to the MERS outbreak. The distribution of respondents at the point of MERS-CoV infection was as follows: patients, 31.7%; healthcare providers, 23.8%; caregivers, 17.5%; and those visiting the patients in hospitals, 17.5% (Table 2). The median length of hospitalization was 21 days (range: 7–120 days), 33% of survivors had pneumonia, 19% received ventilator treatment during MERS, 12.7% had a family member who died from MERS, and 66.7% received financial support.

**Table 1:** Sociodemographic characteristics of participants in MERS survivors, South Korea

Characteristics	Measure	Total (*N* = 63)	
		N or mean	% or SD
Gender	Male	39	61.9
	Female	24	38.1
Age (years)	Total	49.2	12.6
	20–39	17	26.3
	40–49	17	27
	50–59	15	23.8
	60-	14	22.2
Resident area	Seoul (The capital)	21	33.3
	The metropolitan area	23	36.5
	Other provinces	19	30.2
Job	Employed	40	63.5
	Self-employed	11	17.5
	Housewife	3	4.8
	Unemployed or retired	3	4.8
	Others	6	9.5
Marriage	Married	50	79.4
	Unmarried	8	12.7
	Divorce or bereaved	5	8.0
Living with children (who are below 13 years old)	Yes	21	33.4
Religion	Yes	44	69.8
Education	Below middle school	11	17.5
	Graduation from high school	19	30.2
	above university	33	52.4
Monthly income (US dollars, *n* = 56)	below 1700	16	25.4
	1700–3500	20	31.7
	above 3500	20	31.7
Medical illness prior to MERS	Yes	20	31.7
Previous history of psychiatric visit	Yes	10	15.9

*MERS*, Middle East respiratory syndrome

**Table 2:** MERS-related clinical information and experiences of participants in MERS survivors, South Korea

Characteristics	Measure	Total (N = 63)	
		N or mean	% or range
Status at the point of infection	Patients	20	31.7
	Health care workers	15	23.8
	Caregivers	11	17.5
	Visitors	11	17.5
	Others	6	9.5
Days of hospitalization^a^		21	7–120
Days from symptoms to confirmed diagnosis		4	1–15
Total days of illness		23	9–150
Pneumonia	Yes	21	33
Ventilator treatment	Yes	12	19
ECMO treatment	Yes	4	6.3
Presence of a family member who died from MERS	Yes	8	12.7
Financial support	Yes	42	66.7
Information support	Yes	14	22.2

*MERS*, Middle East respiratory syndrome, *ECMO*, extracorporeal membrane oxygenation

^a^Three cases did not answer the question

Overall, 54% of the subjects had at least one symptom of PTSD, depression, suicidality, or insomnia that was significantly above the clinical threshold. The mean total score on the IES-R was 25.93 (SD = 20.01), and 42.9% of the subjects had significant PTSD (Table 3). The mean score on the PHQ-9 was 2.49 (SD = 3.53) before infection with MERS-CoV, 13.54 (SD = 8.80) during the infection, and 6.60 (SD = 6.2) at 12 months after the initial infection. Moreover, 27% of the subjects had depression at 12 months post-MERS. Most subjects had a minimum risk of suicidality, but 22.2% showed at least a moderate degree of suicidal risk. Of the survivors, 28% reported significant insomnia at 12 months after the MERS outbreak. During MERS and 12 months post-MERS, all domains of PTSD, anxiety, and depression were more severe, and the quality of life was worse in survivors with current PTSD or depression compared to those without PTSD or depression (*P* < 0.001) (Table S1). However, anxiety and depression prior to MERS did not significantly differ in either comparison. Survivors with PTSD reported higher scores for negative coping strategies compared to those without PTSD (*P* = 0.001).

**Table 3:** Mental health status of participants in MERS survivors, South Korea

Measure	Cut-off values	Prior to MERS^a^	during MERS^a^	At 12 months post-MERS
		No.(%) of cases		
PTSD (IES-R-K)	0–24: within normal limits	−	−	36 (57.1)
	25–39: mild to moderate	−	−	12 (19.0)
	40–59: severe	−	−	9 (14.3)
	60-: very severe	−	−	6 (9.5)
Depression (PHQ-9)	0–4: no	48 (76.2)	13 (20.6)	28 (44.4)
	5–9: mild	10 (15.9)	8 (12.7)	18 (28.6)
	10–19: moderate	5 (7.9)	21 (33.3)	15 (23.8)
	20–27: severe	None	21 (33.3)	2 (3.2)
Anxiety (GAD-7)	0–4: no	51 (81.0)	19 (30.2)	43 (68.3)
	5–9: mild	7 (11.1)	11 (17.5)	11 (17.5)
	10–14: moderate	4 (6.3)	9 (14.3)	6 (9.5)
	15–21: severe	1 (1.6)	24 (38.1)	3 (4.8)
Suicidality (MINI) ^b^	1–5: low	−	−	46 (76.7)
	6–9: moderate	−	−	7 (11.1)
	10-: high	−	−	7 (11.1)
Insomnia (ISI-K)	> = 15	−	−	18 (28.6)

*MERS*, Middle East respiratory syndrome, *PTSD*, Posttraumatic stress disorder, *IES-R-K*, the Impact of Event Scale-Revised Korean version, *PHQ-9*, the Patient Health Questionnaire-9, *GAD-7*, the Generalized Anxiety Disorder-7, *MINI*, the Mini-International Neuropsychiatric Interview, *ISI*, the Korean version of Insomnia Severity Index

^a^The data were acquired at 12 month by recall

^b^Three of cases showed no response on the questionnaire regarding suicidality

Univariate and multivariate logistic regression analyses were performed to identify risk factors associated with PTSD or depression at 12 months post-MERS. The univariate analysis revealed that several factors were significantly associated with PTSD, including previous psychiatry history, having a family member who died from MERS, depression and anxiety during the MERS-affected period, greater perceived stigma currently and during the illness, and negative coping strategies (Table S2). Depression was associated with gender, previous psychiatry history, anxiety before MERS, having a family member who died from MERS, and depression, anxiety, and greater stigma during the affected phase. Neither the severity of MERS nor complications, such as the development of pneumonia, use of a ventilator, or extracorporeal membrane oxygenation was associated with PTSD or depression. Likewise, not having a spouse, living with a child, and poor social support were not associated with these outcomes.

The multivariate logistic regression analysis revealed that previous psychiatric history (adjusted odds ratio [aOR]: 9.09, 95% confidence interval [CI]: 1.05–78.67; *P* = 0.045), anxiety (aOR: 5.76, 95% CI: 1.29–25.58; *P* = 0.021), and greater recognition of stigma (aOR: 11.09, 95% CI: 2.28–53.90; *P* = 0.003) during the MERS-affected period were independent predictors of PTSD at 12 months after MERS (Table 4). Additionally, previous psychiatric history (aOR: 9.97, 95% CI: 1.53–65.11; *P* = 0.016) and having a family member who died from MERS (aOR: 12.08, 95% CI: 1.47–99.19; *P* = 0.020) predicted the development of depression at this timepoint.

**Table 4:** Multivariate analysis assessing PTSD and depression and related variables 12 months after the MERS outbreak, South Korea

Variables	PTSD^a^	*P*-value	Depression^b^	P-value
	adjusted OR (95% CI)		adjusted OR (95%CI)	
Previous visit to psychiatric clinic				
Yes	9.09 (1.05–78.67)	0.045	9.97 (1.53–65.11)	0.016
No (Ref)	1.00		1.00	
Presence of a family member who died from MERS				
Yes	–	–	12.08 (1.47–99.19)	0.020
No (Ref)	–	–	1.00	
Anxiety during MERS				
Yes	5.76 (1.29–25.58)	0.021	–	–
No (Ref)	1.00		–	–
MERS Stigma during MERS				
High	11.09 (2.28–53.90)	0.003	–	–
Low (Ref)	1.00		–	–

*MERS*, Middle East respiratory syndrome; *PTSD*, Posttraumatic stress disorder; *OR*, odds ratio; *CI*, confidential interval; *Ref*, reference.

^a^Adjusting for presence of previous visit to psychiatric clinic, presence of a family member who died from MERS, anxiety during MERS (GAD> = 10), MERS stigma during MERS, and negative coping

^b^Adjusting for gender, presence of previous visit to psychiatric clinic, presence of a family member who died from MERS, anxiety prior to MERS (GAD> = 10), anxiety during MERS (GAD> = 10), MERS stigma during MERS

## Discussion

The MERS outbreak in 2015 is a noteworthy example of a national disaster that impacted most Korean people. Its early and rapid dissemination via hospitals concentrated in metropolitan areas [2], high fatality rate of nearly 20% [2], and unfamiliarity as a novel infectious disease [26] may have led to high levels of anxiety and fear about being infected among the public and about death among affected people [7]. The present findings confirmed high prevalence of mental health problems in survivors at the recovery phase after the outbreak.

The prevalence of PTSD in survivors at 12 months post-MERS in the present study was comparable to the rate of 41.7% observed in a study of 63 SARS survivors at 3 months post-discharge from a hospital in Singapore [27] and higher or comparable to the rates of PTSD in patients with HIV (30–35%), adult survivors of a human-made disaster (30–60%) [28], and survivors of a stay in an intensive care unit (14–59%) [29, 30]. This indicates that an EID is not only a serious medical illness but also a psychologically traumatic experience for patients that can result in long-term psychological burdens. Additionally, the result suggests that mental health problems caused by an EID outbreak can continue for a long period. For example, another study showed that 42.5% of SARS survivors in Hong Kong still showed active psychiatric illnesses at 3 years post-SARS infection [31]. Furthermore, a second study demonstrated that 42% of Chinese SARS survivors still experienced PTSD at 4 years post-SARS [13]. Assuming that the experiences of the patients in the MERS outbreak are similar in terms of EIDs, the mental health problems of the MERS survivors in the present study may persist for longer than 12 months. Therefore, a study on mental health outcomes after 12 months post-MERS will be required.

Of the premorbid characteristics of the subjects, only a history of a visit to a psychiatric clinic was independently related to PTSD and depression at 12 months post-MERS, whereas demographic factors, such as gender, age, and level of education were not. On the other hand, high anxiety levels, perceived stigma about MERS, and having a family member who died from MERS predicted the development of PTSD or depression. These findings indicate that the psychological outcomes associated with an EID are mainly affected by factors during the outbreak period. Furthermore, the presence of a physical illness prior to the MERS-CoV infection and the severity of MERS were not associated with PTSD or depression. Thus, psychosocial factors, rather than medical factors, may play an important role during MERS-CoV infection in terms of mental health status. These findings differ from those of a study investigating SARS survivors at 30 months post-infection, which found that the risk factors of PTSD included being female, the pre-SARS presence of chronic medical illness, and the presence of complications caused by SARS treatment [32]. It is possible that the relatively small sample size of the present study was insufficient to statistically identify the influences of demographic characteristics and medical severity on adverse psychological outcomes. However, psychological burdens, such as widespread and extreme fear or feelings of isolation caused by MERS [7], may have outweighed the possible contributions of these other factors. A previous report showing that only a history of mental disease and financial burden are related to anxiety in MERS patients [8] supports this assumption.

The present findings suggest a need for appropriate psychosocial support during infectious outbreaks to reduce psychological distress in patients [1]. Therefore, healthcare professionals who treat these patients should be aware of the risk of developing adverse psychological outcomes during the acute stage of the illness as well as during the follow-up period. In particular, patients with a prior psychiatric history, high levels of psychological distress during the illness, or a negative perception about MERS should be given more attention. Interestingly, on our univariate analysis, we can assume that negative coping strategy such as denial, substance use, and self-blame may affect the development of PTSD. This relationship between negative coping style and PTSD is consistent with the previous findings in natural disaster and infectious disease [33, 34]. It suggests that providing what is a useful coping strategy should be included in psychosocial support for survivors from EID.

Similarly, the governmental strategy for the management of EIDs should include psychosocial support based on group characteristics, risk factors, and severity of distress. The White Paper, ‘MERS 2015,’ issued by the Korean government proposed that the national policy for EIDs should include content for “improving ethical problems and strengthening psychological support in EID control.” [1] The present findings suggest several considerations in this regard. In general, during the early outbreak phase, it is important that effective risk communication is incorporated into the overall strategy to reduce fear among the general public and quarantined people [35]; when developing such a strategy for this phase, it is also important to consider the ethical issues related to patients and quarantined people to minimize stigma [36]. More specifically, due to the high prevalence of mental health problems, routine care for EID patients should include effective psychological support that reflects individual risk factors and the current level of distress. In fact, the central and local Korean governments provided psychological support for quarantined people, patients, and families who had a member die from MERS using designated public mental health care centers and telephone counseling during the outbreak [1]. The core value associated with this program was adequate public accessibility; indeed, rather than rely on the passive provision of information, the program was implemented in a proactive manner [37].

In addition, we should pay attention to stigma as a risk factor amenable to change rather than other psychosocial variables for PTSD in the study. In EID outbreak, the perspective is easily made that an infected patient is regarded as a dangerous vector or perpetrator to spread virus who should be isolated from the society [38]. It can be maintained even after the outbreak [39]. The stigma may produce discrimination and exclusion from a community regardless of medical indications. It would significantly threaten a patient’s mental health and social relationship. Consequently, their life could be influenced in a variety of domains such as residence, occupation and the use of healthcare for a long time [40]. This study showed that reducing stigma can be an effective strategy to ameliorate psychological consequence after an EID. Media and government should respect a patient or quarantined people as a citizen who are suffering and be sensitive to words or actions that might stigmatize a specific person or group. A community and healthcare service need to provide active support for an isolated patient to relieve their burden from the stigma [41].

The present study has several limitations that should be noted. Because this study assessed only 43% of the overall MERS survivors, the results may not reflect the status of all survivors. However, the distributions of the demographic data on age, gender, and area of residence in the present study were similar to those in the official reports for all MERS patients [1]. Second, psychological distress and stigma during the pre-MERS period and during the MERS-CoV infection were evaluated based on participant recall and may not accurately represent the actual status of the subjects. Additionally, the relatively small sample size may have limited the ability to identify risk factors due to low statistical power. However, given that 47.2% of 34 patients reported anxiety using the same scale in a previous study conducted during the isolation period [8], it can be assumed that the subjects in the present study were not likely to overestimate their symptoms during recall. Finally, we assessed only with self-questionnaire that could be considered less accurate than the ratings of a clinician.

## Conclusions

Our study showed that nearly half the assessed MERS survivors experienced significant mental health problems, including PTSD and depression, at 12 months post-MERS. MERS-specific psychosocial distress may influence long-term psychological sequelae. Thus, efforts to control EIDs should include all levels of government and involve the implementation of effective strategies to reduce fear and stigma among the public; they should also enable the provision of adequate psychological support and hospital care for infected people.

## Supplementary information

**Additional file 1 Table S1.** Comparisons of mental health status and related factors between survivors with and without PTSD/depression in South Korea. **Table S2.** Univariate analysis assessing PTSD and depression and related variables 12 months after the MERS outbreak, in South Korea.

## Data Availability

The data obtained from the current study are not publicly available due to the sensitive nature of the study.

## Abbreviations

DSM-IV: Diagnostic and Statistical Manual of Mental Disorders-fourth edition
EID: Emerging Infectious Disease
GAD-7: Generalized Anxiety Disorder-7
HIV: Human Immunodeficiency Virus
IES-R-K: Impact of Event Scale-Revised Korean version
ISI-K: Insomnia Severity Index
MOS-SSS: Medical Outcome Study Social Support Survey
MERS-CoV: Middle East respiratory syndrome coronavirus
PHQ-9: Patient Health Questionnaire-9
PTSD: posttraumatic stress disorder
SARS: Severe Acute Respiratory Syndrome
