# Supplementary material for: Posttraumatic stress disorder and depression of survivors 12 months after the outbreak of Middle East respiratory syndrome in South Korea
Source: BMC Public Health. 2020 May 15;20:605. doi: 10.1186/s12889-020-08726-1 (PMC7224724; doi:10.1186/s12889-020-08726-1)
Supplement: Supplementary file 1 — Additional file 1 Table S1. Comparisons of mental health status and related factors between survivors with and without PTSD/depression in South Korea. Table S2. Univariate analysis assessing PTSD and depression and related variables 12 months after the MERS outbreak, in South Korea. [file 12889_2020_8726_MOESM1_ESM.docx]

**Supplementary files**

**Title: Posttraumatic stress disorder and depression of survivors 12 months after the outbreak of Middle East Respiratory Syndrome in South Korea**

**Authors:** Hye Yoon Park, Wan Beom Park, So Hee Lee, Jeong Lan Kim, Jung Jae Lee, Haewoo Lee, Hyoung-Shik Shin

**Table S1.** **Comparisons of mental health status and related factors between survivors with and without PTSD/depression in South Korea**^*^

| Variables | No Current PTSD  (n=36) | Current  PTSD  (n=27) | t | P-value | No Current Depression (n=46) | Current Depression (n=17) | | t | | P-value | |
| --- | --- | --- | --- | --- | --- | --- | --- | --- | --- | --- | --- |
| PTSD |  |  |  |  |  |  | |  | |  | |
| IES-R-K (total) | 11.8 (7.9) | 44.7 (15.1) | 10.311 | <0.001 | 18.3 (13.8) | 46.5 (19.9) | | -5.383 | | <0.001 | |
| Intrusion | 4.4 (4.1) | 16.3 (6.6) | -8.201 | <0.001 | 6.5 (5.7) | 17.4 (8.0) | | -5.107 | | <0.001 | |
| Avoidance | 5.3 (3.5) | 16.8 (6.4) | -8.458 | <0.001 | 8.0 (6.2) | 16.2 (7.6) | | -4.350 | | <0.001 | |
| Numbness | 2.1 (2.3) | 11.7 (5.2) | -8.979 | <0.001 | 3.7 (3.7) | 12.9 (6.1) | | -5.818 | | <0.001 | |
| Anxiety (GAD-7) |  |  |  |  |  |  |  | |  | |  |
| Prior to MERS | 1.4 (2.6) | 4.0 (5.2) | 2.386 | 0.022 | 1.3 (2.4) | 5.9 (5.7) | | -3.209 | | 0.005 | |
| during MERS | 7.1 (7.0) | 15.3 (5.2) | 5.158 | <0.001 | 8.4 (7.1) | 16.5(4.9) | | -5.054 | | <0.001 | |
| At 12 months, post-MERS | 1.6 (2.1) | 7.4 (5.4) | 5.314 | <0.001 | 1.8 (1.9) | 10.4 (4.8) | | -7.135 | | <0.001 | |
| Depression (PHQ-9) |  |  |  |  |  |  | |  | |  | |
| Prior to MERS | 1.8 (3.3) | 3.4 (3.7) | -1.737 | 0.088 | 1.7 (3.0) | 4.5 (4.0) | | -2.593 | | 0.016 | |
| during MERS | 10.0 (8.7) | 18.2 (6.6) | -4.091 | <0.001 | 11.2 (8.6) | 19.9 (5.8) | | -4.639 | | <0.001 | |
| At 12 months, post-MERS | 3.8 (3.8) | 10.4 (6.7) | -4.588 | <0.001 | 3.6 (2.8) | 14.8 (5.2) | | -8.407 | | <0.001 | |
| Coping strategy (Brief COPE) |  |  |  |  |  |  | |  | |  | |
| Emotional strategy | 23.6 (5.6) | 24.3 (5.3) | -0.504 | 0.616 | 24.0 (5.3) | 23.4 (5.9) | | 0.443 | | 0.659 | |
| Problem-solving | 15.7 (3.7) | 16.6 (3.7) | -0.917 | 0.363 | 16.2 (3.6) | 15.7 (4.0) | | 0.465 | | 0.643 | |
| Negative strategy | 19.9 (4.4) | 24.0 (5.3) | -3.371 | 0.001 | 20.7 (4.3) | 24.2 (6.6) | | -2.471 | | 0.016 | |
| Social support |  |  |  |  |  |  | |  | |  | |
| MOS-SSS (total) | 72.9 (13.7) | 67.4 (18.7) | 1.311 | 0.197 | 73.0 (14.6) | 63.9 (18.5) | | 2.032 | | 0.046 | |
| Emotional | 28.8 (6.9) | 26.3 (9.2) | 1.252 | 0.215 | 28.8 (7.1) | 24.8 (9.7) | | 1.833 | | 0.072 | |
| Tangible | 15.5 (4.0) | 15.7 (4.4) | -0.217 | 0.829 | 15.7 (3.8) | 15.2 (5.1) | | 0.388 | | 0.699 | |
| Social | 12.6 (2.4) | 11.1 (3.9) | 1.751 | 0.109 | 12.4 (2.8) | 10.6 (4.0) | | 1.714 | | 0.100 | |
| Affectionate | 12.3 (2.8) | 10.6 (4.3) | 1.814 | 0.094 | 12.2 (3.0) | 9.8 (4.5) | | 2.030 | | 0.055 | |

^*^PTSD, Posttraumatic stress disorder; IES-R-K, the Impact of Event Scale-Revised Korean version; GAD-7, the Generalized Anxiety Disorder-7; PHQ-9, the Patient Health Questionnaire-9; MOS-SSS, the Medical Outcome Study Social Support Survey.

**Table S2.** **Univariate analysis assessing PTSD and depression and related variables 12 months after the MERS outbreak, in South Korea**^*^

|  | No Current PTSD  (n=36) | | Current  PTSD  (n=27) | | | | Odds Ratio  (95%CI) | | | | | P-value | | | No Current Depression (n=46) | | | | | Current Depression (n=17) | | | | | | | Odds Ratio  (95%CI) | | | | | | | | | P-value | | |  |  |  |  |  |
| --- | --- | --- | --- | --- | --- | --- | --- | --- | --- | --- | --- | --- | --- | --- | --- | --- | --- | --- | --- | --- | --- | --- | --- | --- | --- | --- | --- | --- | --- | --- | --- | --- | --- | --- | --- | --- | --- | --- | --- | --- | --- | --- | --- |
| Gender | |  | |  | | | |  | | | | |  | | | |  | | | | |  | | | | | | |  | | | | | | | |  | | | |  |  |  |
| M (Ref) | | 23 (63.9) | | 16 (59.3) | | | | 1.00 | | | | |  | | | | 32 (69.6) | | | | | 7 (41.2) | | | | | | | 1.00 | | | | | | | |  | | | |  |  |  |
| F | | 13 (36.1) | | 11 (40.7) | | | | 1.22 (0.42-3.39) | | | | | 0.708 | | | | 14 (30.4) | | | | | 10 (58.8) | | | | | | | 3.27 (1.03-10.33) | | | | | | | | 0.039 | | | |  |  |  |
| Age | |  | |  | | | |  | | | | |  | | | |  | | | | |  | | | | | | |  | | | | | | | |  | | | |  |  |  |
| <50 years (Ref) | | 19 (52.8) | | 15 (55.6) | | | | 1.00 | | | | |  | | | | 27 (58.7) | | | | | 7 (41.2) | | | | | | | 1.00 | | | | | | | |  | | | |  |  |  |
| >=50 years | | 17 (47.2) | | 12 (44.4) | | | | 0.89 (0.33-2.44) | | | | | 0.827 | | | | 19 (41.3) | | | | | 10 (58.8) | | | | | | | 2.03 (0.66-6.29) | | | | | | | | 0.216 | | | |  |  |  |
| Education | |  | |  | | | |  | | | | |  | | | |  | | | | |  | | | | | | |  | | | | | | | |  | | | |  |  |  |
| <= high school (Ref) | | 18 (50.0) | | 12 (44.4) | | | | 1.00 | | | | |  | | | | 23 (50.0) | | | | | 7 (41.2) | | | | | | | 1.00 | | | | | | | |  | | | |  |  |  |
| >=university | | 18 (50.0) | | 15 (55.6) | | | | 1.25 (0.46-3.40) | | | | | 0.662 | | | | 23 (50.0) | | | | | 10 (58.8) | | | | | | | 1.43 (0.46-4.40) | | | | | | | | 0.239 | | | |  |  |  |
| Having a spouse | |  | |  | | | |  | | | | |  | | | |  | | | | |  | | | | | | |  | | | | | | | |  | | | |  |  |  |
| Yes (Ref) | | 7 (19.5) | | 6 (22.2) | | | | 1.00 | | | | |  | | | | 38 (82.6) | | | | | 12 (70.6) | | | | | | | 1.00 | | | | | | | |  | | | |  |  |  |
| No | | 29 (80.6) | | 21 (77.8) | | | | 0.85 (0.25-2.88) | | | | | 0.787 | | | | 8 (17.4) | | | | | 5 (29.4) | | | | | | | 1.98 (0.54-7.21) | | | | | | | | 0.295 | | | |  |  |  |
| Religion | |  | |  | | | |  | | | | |  | | | |  | | | | |  | | | | | | |  | | | | | | | |  | | | |  |  |  |
| Yes (Ref) | | 13 (36.1) | | 6 (22.2) | | | | 1.00 | | | | |  | | | | 31 (67.4) | | | | | 13 (76.5) | | | | | | | 1.00 | | | | | | | |  | | | |  |  |  |
| No | | 23 (63.9) | | 21 (77.8) | | | | 1.98 (0.64-6.15) | | | | | 0.235 | | | | 15 (32.6) | | | | | 4 (23.5) | | | | | | | 0.64 (0.18-2.29) | | | | | | | | 0.486 | | | |  |  |  |
| Monthly income (US dollars) | | | | | |  | | |  | | | | |  | | | | |  | | | | | | | | | | | | |  |  |  |  |  |  |  |  |  |  |  |  |
| Above 3500 (Ref) | | 17 (48.6) | | 16 (61.5) | | | | 1.00 | | | | |  | | | | 22 (50.0) | | | | | 11 (64.7) | | | | | | | 1.00 | | | | | | | |  | | | |  |  |  |
| Below 3500 | | 18 (51.4) | | 10 (38.5) | | | | 0.59 (0.21-1.66) | | | | | 0.315 | | | | 22 (50.0) | | | | | 6 (35.3) | | | | | | | 0.55 (0.17-1.74) | | | | | | | | 0.301 | | | |  |  |  |
| Living with a child | |  | |  | | | |  | | | | |  | | | |  | | | | |  | | | | | | |  | | | | | | | |  | | | |  |  |  |
| No (Ref) | | 23 (63.9) | | 19 (70.4) | | | | 1.00 | | | | |  | | | | 30 (65.2) | | | | | 12 (70.6) | | | | | | | 1.00 | | | | | | | |  | | |  |  |  |  |
| Yes | | 13 (36.1) | | 8 (29.6) | | | | 0.75 (0.26-2.17) | | | | | 0.589 | | | | 16 (34.8) | | | | | 5 (29.4) | | | | | | | 0.78 (0.23-2.61) | | | | | | | | 0.688 | | |  |  |  |  |
| Previous medical illness | | | | |  | | | | |  | | | | | |  | | | | |  | | | | | | |  | | | | | | |  | | | | | | | | |
| No (Ref) | | 23 (63.9) | | 20 (74.1) | | | | 1.00 | | | | |  | | | | 31 (67.4) | | | | | 12 (70.6) | | | | | | | 1.00 | | | | | | | |  | | |  |  |  |  |
| Yes | | 13 (36.1) | | 7 (25.9) | | | | 0.62 (0.21-1.86) | | | | | 0.390 | | | | 15 (32.6) | | | | | 5 (29.4) | | | | | | | 0.86 (0.26-2.89) | | | | | | | | 0.809 | | |  |  |  |  |
| Previous visit to psychiatric clinic | | | | | |  | | |  | | | | |  | | | | |  | | | | | | | | | | | | |  |  |  |  |  |  |  |  |  |  |  |  |
| No (Ref) | | 34 (94.4) | | 19 (70.4) | | | | 1.00 | | | | |  | | | | 43 (93.5) | | | | | 10 (58.8) | | | | | | | 1.00 | | | | | | | |  | | |  |  |  |  |
| Yes | | 2 (5.6) | | 8 (29.6) | | | | 7.16 (1.38-37.20) | | | | | 0.014 | | | | 3 (6.5) | | | | | 7 (41.2) | | | | | | | 10.03 (2.20-45.76) | | | | | | | | 0.003 | | |  |  |  |  |
| Status at the point of infection | | | | | |  | | |  | | | | |  | | | | |  | | | | | | | | | | | | |  |  |  |  |  |  |  |  |  |  |  |  |
| No HCW (Ref) | | 27 (75.0) | | 21 (77.8) | | | | 1.00 | | | | |  | | | | 33 (71.7) | | | | | 15 (88.2) | | | | | | | 1.00 | | | | | | | |  | | |  |  |  |  |
| HCW | | 9 (25.0) | | 6 (22.2) | | | | 0.86 (0.26-2.79) | | | | | 0.798 | | | | 13 (32.5) | | | | | 2 (11.8) | | | | | | | 0.34 (0.07-1.69) | | | | | | | | 0.317 | | |  |  |  |  |
| Duration of hospitalization | | | | | |  | | |  | | | | |  | | | | |  | | | | | | | | | | | | |  |  |  |  |  |  |  |  |  |  |  |  |
| Low 75^th^ (Ref) | | 23 (91.7) | | 18 (66.7) | | | | 1.00 | | | | |  | | | | 40 (87.0) | | | | | 11 (64.7) | | | | | | | 1.00 | | | | | | | |  | | |  |  |  |  |
| High 25^th^ | | 3 (8.3) | | 9 (35.3) | | | | 5.50 (1.32-22.92) | | | | | 0.012 | | | | 6 (13.0) | | | | | 6 (35.3) | | | | | | | 3.64(0.98-13.53) | | | | | | | | 0.046 | | |  |  |  |  |
| Pneumonia | |  | |  | | | |  | | | | |  | | | |  | | | | |  | | | | | | |  | | | | | | | |  | | |  |  |  |  |
| No (Ref) | | 26 (72.2) | | 16 (59.3) | | | |  | | | | |  | | | | 32 (69.6) | | | | | 10 (58.8) | | | | | | |  | | | | | | | |  | | |  |  |  |  |
| Yes | | 10 (27.8) | | 11 (40.7) | | | | 1.79 (0.62-5.15) | | | | | 0.280 | | | | 14 (30.4) | | | | | 7 (41.2) | | | | | | | 1.60 (0.51-5.06) | | | | | | | | 0.422 | | |  |  |  |  |
| Ventilator treatment | |  | |  | | | |  | | | | |  | | | |  | | | | |  | | | | | | |  | | | | | | | |  | | |  |  |  |  |
| No (Ref) | | 32 (88.9) | | 19 (70.4) | | | |  | | | | |  | | | | 38 (82.6) | | | | | 13 (76.5) | | | | | | |  | | | | | | | |  | | |  |  |  |  |
| Yes | | 4 (11.1) | | 8 (29.6) | | | | 3.37 (0.89-12.71) | | | | | 0.104 | | | | 8 (17.4) | | | | | 4 (23.5) | | | | | | | 1.46 (0.38-5.67) | | | | | | | | 0.582 | | |  |  |  |  |
| ECMO treatment | |  | |  | | | |  | | | | |  | | | |  | | | | |  | | | | | | |  | | | | | | | |  | | |  |  |  |  |
| No (Ref) | | 34 (94.4) | | 25 (92.6) | | | |  | | | | |  | | | | 43 (93.5) | | | | | 16 (94.1) | | | | | | |  | | | | | | | |  | | |  |  |  |  |
| Yes | | 2 (5.6) | | 2 (7.4) | | | | 1.36 (0.18-10.32) | | | | | 1.000 | | | | 3 (6.5) | | | | | 1 (5.9) | | | | | | | 0.90 (0.09-9.25) | | | | | | | | 1.000 | | |  |  |  |  |
| Presence of a family member who died from MERS | | | | | | | | | | |  | | | | | | | |  | | | | |  |  |  |  |  |  |  |  |  |  |  |  |  |  |  |  |  |  |  |  |
| No (Ref) | | 34 (94.4) | | 21 (77.8) | | | |  | | | | |  | | | | 43 (93.5) | | | | | 12 (70.6) | | | | | | |  | | | | | | | |  | | | |  |  |  |
| Yes | | 2 (5.6) | | 6 (22.2) | | | | 4.86 (0.90-26.33) | | | | | 0.065 | | | | 3 (6.5) | | | | | 5 (29.4) | | | | | | | 5.97 (1.25-28.6) | | | | | | | | 0.028 | | | |  |  |  |
| Financial support | |  | |  | | | |  | | | | |  | | | |  | | | | |  | | | | | | |  | | | | | | | |  | | | |  |  |  |
| Yes (Ref) | | 27 (75.0) | | 15 (55.6) | | | |  | | | | |  | | | | 31 (67.4) | | | | | 11(64.7). | | | | | | |  | | | | | | | |  | | | |  |  |  |
| No | | 9 (25.0) | | 12 (44.4) | | | | 2.40 (0.82-7.00) | | | | | 0.105 | | | | 15 (32.6) | | | | | 6 (35.3) | | | | | | | 1.13 (0.35-3.63) | | | | | | | | 0.841 | | | |  |  |  |
| Information support | |  | |  | | | |  | | | | |  | | | |  | | | | |  | | | | | | |  | | | | | | | |  | | | |  |  |  |
| Yes (Ref) | | 10 (27.8) | | 4 (15.4) | | | |  | | | | |  | | | | 10 (21.7) | | | | | 4 (25.0) | | | | | | |  | | | | | | | |  | | | |  |  |  |
| No | | 26 (72.2) | | 22 (84.6) | | | | 2.12 (0.58-7.69) | | | | | 0.249 | | | | 36 (78.3) | | | | | 12 (75.0) | | | | | | | 0.83 (0.22-3.154) | | | | | | | | 0.743 | | | |  |  |  |
| Anxiety, premorbid | |  | |  | | | |  | | | | |  | | | |  | | | | |  | | | | | | |  | | | | | | | |  | | | |  |  |  |
| No (Ref) | | 35 (97.2) | | 23 (85.2) | | | |  | | | | |  | | | | 45 (97.8) | | | | | 13 (76.5) | | | | | | |  | | | | | | | |  | | | |  |  |  |
| Yes | | 1 (2.8) | | 4 (14.8) | | | | 6.01 (0.64-57.96) | | | | | 0.155 | | | | 1 (2.2) | | | | | 4 (23.5) | | | | | | | 13.85 (1.42-134.90) | | | | | | | | 0.016 | | | |  |  |  |
| Depression, premorbid | | | | |  | | | | |  | | | | | |  | | | | |  | | | | | | |  | | | | | | |  | | | | | | | | |
| No (Ref) | | 34 (94.4) | | 24 (88.9) | | | |  | | | | |  | | | | 44 (95.7) | | | | | 14 (82.4) | | | | | | |  | | | | | | | |  | | | |  |  |  |
| Yes | | 2 (5.6) | | 3 (11.1) | | | | 2.13 (0.33-13.70) | | | | | 0.643 | | | | 2 (4.3) | | | | | 3 (17.6) | | | | | | | 4.71 (0.71-31.13) | | | | | | | | 0.117 | | | |  |  |  |
| Anxiety during MERS | | | | |  | | | | |  | | | | | |  | | | | |  | | | | | | |  | | | | | | |  | | | | | | | | |
| No (Ref) | | 25 (69.4) | | 5 (18.5) | | | |  | | | | |  | | | | | 27 (58.7) | | | | | 3 (17.6) | | | | | | | |  | | | | | | |  | | | |  |  |
| Yes | | 11 (30.6) | | 22 (81.5) | | | | 10.00 (3.01-33.29) | | | | | <0.001 | | | | | 19 (41.3) | | | | | 14 (82.4) | | | | | | | | 6.63 (1.67-26.31) | | | | | | | 0.005 | | | |  |  |
| Depression during MERS | | | | |  | | | | |  | | | | | |  | | | | |  | | | | |  | | | | | | | |  | | | | | | | | |  |
| No (Ref) | | 17 (47.2) | | 4 (14.8) | | | |  | | | | |  | | | | | 20 (43.5) | | | | | 1 (5.9) | | | | | | | |  | | | | | | |  | | | |  |  |
| Yes | | 19 (52.8) | | 23 (85.2) | | | | 5.15 (1.48-17.91) | | | | | 0.008 | | | | | 26 (56.5) | | | | | 16 (94.1) | | | | | | | | 12.31 (1.50-100.78) | | | | | | | 0.006 | | | |  |  |
| MERS Stigma during MERS | | | | | |  | | |  | | | | |  | | | | |  | | | | | |  | | | | |  |  |  |  |  |  |  |  |  |  |  |  |  |  |
| Low (Ref) | | 27 (75.0) | | 7 (20.6) | | | |  | | | | |  | | | | | 29 (63.0) | | | | | 5 (29.4) | | | | | | | |  | | | | | | |  | | | |  |  |
| High | | 9 (25.0) | | 20 (74.1) | | | | 8.57 (2.73-26.92) | | | | | <0.001 | | | | | 17 (37.0) | | | | | 12 (70.6) | | | | | | | | 4.09 (1.23-13.63) | | | | | | | 0.017 | | | |  |  |
| Current MERS Stigma | |  | | |  | | | | |  | | | | | |  | | | | |  | | | | | | |  | | | | | | |  | | | | | | | | |
| Low (Ref) | | 22 (61.1) | | 9 (34.6) | | | |  | | | | |  | | | | 24 (52.2) | | | | | 7 (43.8) | | | | | | |  | | | | | | | |  | | | |  |  |  |
| High | | 14 (38.9) | | 17 (65.4) | | | | 2.97 (1.04-8.45) | | | | | 0.039 | | | | 22 (47.8) | | | | | 9 (56.2) | | | | | | | 1.40 (0.45-4.41) | | | | | | | | 0.562 | | | |  |  |  |
| Emotional coping | |  | |  | | | |  | | | | |  | | | |  | | | | |  | | | | | | |  | | | | | | | |  | | | |  |  |  |
| Low (Ref) | | 19 (52.8) | | 13 (48.1) | | | |  | | | | |  | | | | 23 (50.0) | | | | | 8 (47.1) | | | | | | |  | | | | | | | |  | | | |  |  |  |
| High | | 17 (47.2) | | 14 (51.9) | | | | 1.20 (0.44-3.27) | | | | | 0.716 | | | | 23 (50.0) | | | | | 9 (52.9) | | | | | | | 1.13 (0.37-3.43) | | | | | | | | 0.836 | | | |  |  |  |
| Problem solving coping | | | | |  | | | | |  | | | | | |  | | | | |  | | | | | | |  | | | | | | |  | | | | | | | | |
| Low (Ref) | | 20 (55.6) | | 12 (44.4) | | | |  | | | | |  | | | | 24 (52.2) | | | | | 7 (41.2) | | | | | | |  | | | | | | | |  | | | |  |  |  |
| High | | 16 (44.4) | | 15 (55.6) | | | | 1.56 (0.57-4.27) | | | | | 0.383 | | | | 22 (47.8) | | | | | 10 (58.8) | | | | | | | 1.56 (0.51-4.81) | | | | | | | | 0.438 | | | |  |  |  |
| Negative coping | |  | |  | | | |  | | | | |  | | | |  | | | | |  | | | | | | |  | | | | | | | |  | | | |  |  |  |
| Low (Ref) | | 24 (66.7) | | 10 (37.0) | | | |  | | | | |  | | | | 27 (58.7) | | | | | 7 (41.2) | | | | | | |  | | | | | | | |  | | | |  |  |  |
| High | | 12 (33.3) | | 17 (63.0) | | | | 3.40 (1.20-9.66) | | | | | 0.020 | | | | 19 (41.3) | | | | | 10 (58.8) | | | | | | | 2.03 (0.66-6.29) | | | | | | | | 0.216 | | | |  |  |  |
| Social support | |  | |  | | | |  | | | | |  | | | |  | | | | |  | | | | | | |  | | | | | | | |  | | | |  |  |  |
| Low 25th (Ref) | | 7 (19.4) | | 9 (33.3) | | | |  | | | | |  | | | | 10 (21.7) | | | | | 6 (35.3) | | | | | | |  | | | | | | | |  | | | |  |  |  |
| High 75th | | 29 (80.6) | | 18 (66.7) | | | | 2.07 (0.66-6.54) | | | | | 0.210 | | | | 36 (78.3) | | | | | 11(64.7). | | | | | | | 1.96 (0.58-6.63) | | | | | | | | 0.273 | | | |  |  |  |

^*^MERS, Middle East Respiratory Syndrome; PTSD, Posttraumatic stress disorder; CI, confidential interval; Ref, reference. N.S, non-significant; P-value was estimated by chi-square test or Fisher’s exact test in some cases that consists of cells < 5%.
